# Single-Electrode Electrostatic Repulsion Phenomenon for Remote Actuation and Manipulation

**DOI:** 10.34133/research.0393

**Published:** 2024-05-29

**Authors:** Wei Tang, Dong Yan, Kecheng Qin, Xinyu Guo, Yiding Zhong, Huxiu Xu, Huayong Yang, Jun Zou

**Affiliations:** ^1^ State Key Laboratory of Fluid Power and Mechatronic Systems, Zhejiang University, Hangzhou, China.; ^2^ School of Mechanical Engineering, Zhejiang University, Hangzhou, China.; ^3^ Institute of Process Equipment, College of Energy Engineering, Zhejiang University, Hangzhou, China.

## Abstract

One of the fundamental principles of electrostatics is that an uncharged object will be attracted to a charged object through electrostatic induction as the two approaches one another. We refer to the charged object as a single electrode and examine the scenario where a positive voltage is applied. Because of electrostatic induction phenomenon, single-electrode electrostatics only generates electrostatic attraction forces. Here, we discover that single-electrode electrostatics can generate electrostatic repulsion forces and define this new phenomenon as single-electrode electrostatic repulsion phenomenon. We investigate the fundamental electrostatic phenomena, giving a curve of electrostatic force versus voltage and then defining 3 regions. Remote actuation and manipulation are essential technologies that are of enormous concern, with tweezers playing an important role. Various tweezers designed on the basis of external fields of optics, acoustics, and magnetism can be used for remote actuation and manipulation, but some inherent drawbacks still exist. Tweezers would benefit greatly from our discovery in electrostatics. On the basis of this discovery, we propose the concept of electrostatic tweezers, which can achieve noncontact and remote actuation and manipulation. Experimental characterizations and successful applications in metamaterials, robots, and manipulating objects demonstrated that electrostatic tweezers can produce large deformation rates (>6,000%), fast actuation (>100 Hz), and remote manipulating distance (~15 cm) and have the advantages of simple device structure, easy control, lightweight, no dielectric breakdown, and low cost. Our work may deepen people’s understanding of single-electrode electrostatics and opens new opportunities for remote actuation and manipulation.

## Introduction

Electrostatics [1] is ubiquitous in our life and plays an essential role across a diverse range of topics in physics, electronics, chemistry, biology, medicine, agriculture, and industry [2]. As a result, electrostatics has been extensively studied and applied in the past centuries [3]. In electrostatics, it is well known that electrostatic induction would generate electrostatic attraction phenomenon [4–6], i.e., an electrically neutral object would be attracted to the electrode with electrostatic charge, where this electrode with electrostatic charge could be termed as a single electrode. However, in our recent experiments, there were some anomalies. When we reproduced the electrostatic induction, we observed that the single electrode generated the typical electrostatic attraction phenomenon when the voltage was low; and yet, when the voltage increased and exceeded a specific threshold value, the object that was supposed to be attracted were repelled (Movies S1 and S2).

We carried out a series of studies on this anomalous phenomenon and defined it as single-electrode electrostatic repulsion phenomenon. Using a basic test setup that consisted of a single-electrode sheet and a deformation sheet, we investigated the fundamental electrostatic phenomena, giving a curve of electrostatic force versus voltage and then defining 3 regions: electrostatic attraction region, coexistence region, and electrostatic repulsion region. We use theoretical analysis, simulation, and experiment methods to explain the phenomenon.

Remote actuation [7,8] and manipulation [9] are widely used in scientific research, industrial production, aerospace, and medical devices [10–12], where various tweezers created using external physical fields such as optics [13], acoustics [14], and magnetism [15–17] can be used for remote actuation and manipulation. Proposed in 1986, optical tweezers [18,19] are capable of manipulating particles with sizes ranging from tens of nanometers to tens of micrometers. Although they are frequently used to work with atoms, molecules, and biological cells, this method is limited to working with tiny objects. Acoustic tweezers [20,21] use sound waves to control the movement of small objects and are widely used for manipulating cells and nanomaterials; however, this method requires a closed space to be used. Magnetic actuation [22,23] finds extensive application in medical examinations and micro/nanorobots; nevertheless, the things it operates and actuates need to be composed of or include ferromagnetic elements. Using tweezers to remotely actuate and manipulate various macroscopic objects in real-world circumstances is a persistent challenge. Electrostatic method [24–29] can be used to actuate and manipulate various objects, mostly by electrostatic attraction [5], such as manipulating droplets via electrostatic induction [30]. However, this method has a very restricted range of action, easily attracts objects to the electrode, and cannot achieve remote actuation and manipulation.

Single-electrode electrostatic repulsion phenomenon that we discovered opens up the possibility for the implementation of remote electrostatic actuation and manipulation. We propose the concept of electrostatic tweezers, which can achieve noncontact and remote actuation and manipulation. Electrostatic tweezers are composed of a single electrode or a combination of multiple single electrodes and can be used for actuation and manipulation of various objects, including conductors and nonconductors. We use electrostatic tweezers for actuation, which can be utilized for noncontact and remote actuating of deformable sheets, producing large deformation rates (>6,000%) and fast actuation (>100 Hz) in the field of smart material actuation. Compared with the traditional electrostatic actuation methods [31–37], electrostatic tweezers not only are free of fatal defects such as dielectric breakdown or short circuit but also have a simple structure, extremely lightweight, and large deformation, showing a wide range of application prospects in metamaterials and robots. In addition, to verify manipulation capability of electrostatic tweezers, we used them to manipulate macroscopic-sheet-like and spherical objects, enabling fast and multidimensional remote manipulation (distance, ~15 cm). Compared with optical [13,18,19], acoustic [14,20,21], and magnetic [15–17,22,23] tweezers, electrostatic tweezers have the advantages of simple device and wide range of operating objects, providing unprecedented opportunities for remote actuation and manipulation various macroscopic objects in real-world circumstances.

## Results

### Single-electrode electrostatic repulsion phenomenon

**Fig. 1.**
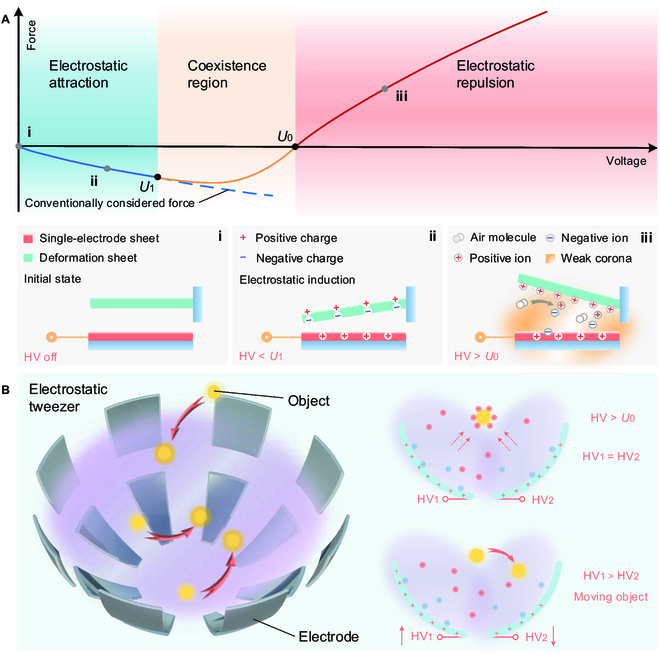
Single-electrode electrostatic repulsion phenomenon and concept of electrostatic tweezer. (A) Electrostatic force versus voltage curve for single-electrode electrostatics. The curve is divided into 3 regions: electrostatic attraction region, coexistence region, and electrostatic repulsion region. *U*_1_ and *U*_0_ are coexistence voltage threshold and switching voltage threshold, respectively: (i) is initial state, (ii) is schematic of electrostatic attraction, and (iii) is schematic of electrostatic repulsion. HV, high voltage. (B) Concept of electrostatic tweezer.

To discuss this phenomenon, we built a basic test setup following the conventional electrostatic induction experiments, which consisted of a single-electrode sheet (electrostatic tweezer) and a deformation sheet (object) (Fig. 1A, Fig. S1, Movie S2, and Materials and Methods). When the voltage applied to the electrode sheet was gradually increased, we found that the bending angle of the deformation sheet being attracted gradually became larger as the voltage increased below *U*_1_, i.e., the electrostatic attraction force gradually became larger as voltage increased. When the voltage exceeded *U*_1_, the angle at which the deformation sheet is attracted became smaller instead, i.e., the electrostatic attraction force gradually became smaller as voltage increased. When the voltage continued to increase beyond *U*_0_, the deformation sheet was no longer attracted but rather repelled. Thereafter, as the voltage increased, the deployable deformation gradually increased with voltage, which meant that the electrostatic repulsion gradually became larger as voltage increased. We define the voltage value at which the electrostatic attraction force begins to become smaller as *U*_1_, i.e., coexistence voltage threshold, the voltage at which the electrostatic force switches from attraction to repulsion as *U*_0_, i.e., switching voltage threshold, the range from 0 to *U*_1_ as the electrostatic attraction region, the range from *U*_1_ to *U*_0_ as the coexistence region, and the range above *U*_0_ as the electrostatic repulsion region (Fig. 1A).

To explore the mechanism underlying this phenomenon, we meticulously analyzed the discharge process on the single-electrode sheet. When the voltage is below *U*_1_, it is the conventional electrostatic induction phenomenon. When the voltage exceeds *U*_0_, we believe that the electrode produces a weak corona phenomenon that deprives some air molecules around the electrode of electrons, converting them into positive ions, which then move in the space (analysis can be found in the “Vacuum test” section). At this moment, some positive ions would attach to the deformation sheet, causing it to appear macroscopically as positively charged rather than electrically neutral. Under the action of Coulomb force, the deformation sheet would be repelled by the single-electrode sheet because particles with same electric charges repel each other. When the voltage is reduced below *U*_0_, the corona phenomenon weakens, the electrostatic induction phenomenon regains the upper hand, and the deformation sheet is attracted; while increasing the voltage above *U*_0_, the deformation sheet is repelled again. This phenomenon can achieve continuous reversible switching of attraction and repulsion forces by adjusting the magnitude of the voltage. It is worth noting that when the voltage is reduced compared to before the charges are attached, the attraction force produced by the same voltage is smaller, and *U*_0_ also changes since there are some charges attached to the deformation sheet under high voltage. This effect weakens as the area of the deformation sheet increases and as the time interval grows, i.e., the faster the object to which the charge is attached returns to electrical neutrality, the smaller the effect. Detailed theoretical analysis and simulation of this phenomenon is presented in the next section, “Theoretical analysis and simulation”.

The discovery of this phenomenon opens up the possibility of using electrostatic methods for remote actuation and manipulation. We propose the concept of electrostatic tweezers, which are composed of a single electrode or a combination of multiple single electrodes. A single-electrode tweezer can be used for actuation, requiring only one deformation sheet. Figure 1B shows the multielectrode electrostatic tweezers that can manipulate objects by controlling several electrodes with voltage above *U*_0_. There are no special requirements for the material of the object being manipulated in electrostatic tweezers, which can be a conductor or a nonconductor (Fig. S2, Movie S3, and the “Electrostatic tweezer drives various materials” section), such as copper film, paper, wood, plastic, etc. Our electrostatic tweezers have the advantages of simple device structure, easy control, and low cost, making them suitable for remote actuation and manipulation of macroscopic objects.

### Theoretical analysis and simulation

Single-electrode electrostatic repulsion phenomenon is caused by a weak corona, and the repulsion force is the result of ion attachment and Coulomb forces. With reference to the relevant corona theory [38–40], we conduct theoretical analysis and numerical simulation of this phenomenon. When exposed to air, high-voltage electrodes ionize the surrounding air, resulting in the corona phenomenon at a specified voltage range. Corona can cause a large number of free positive ions to be generated in the air around the electrode. We can use a self-consistent plasma model to calculate the ion concentration around the electrode at a specific voltage, where the model assumes a one-dimensional problem.

Neglecting the electron convection due to air motion, the electron density can be calculated by the drift-diffusion equation as

$$\frac{\partial }{\partial t}\left(n_{e}\right)+\nabla \cdot \left[-n_{e}\left(\mu_{e}\cdot E\right)-D_{e}\cdot \nabla n_{e}\right]=R_{e}$$ (1)

The source coefficients in Eq. 1 are related to the chemical composition of the plasma. Assuming that there are *M* reactions during the ionization process connected to variations in electron density, and for DC discharges using Townsend coefficients, the electron source term can be expressed as

$$R_{e}=\sum_{j=1}^{M}x_{j}\alpha_{j}N_{n}\;|\;\Gamma_{e}\;|$$ (2)

where *x_j_* is the molar fraction, *α_j_* is the Townsend coefficient of reaction *j*, *N_n_* is the total neutral number density, and *Г_e_* is the electron flux.

The electron mobility is

$$D_{e}=\mu_{e}T_{e}$$ (3)

The mass fraction of each substance is

$$\rho \frac{\partial }{\partial t}\left(w_{k}\right)+\rho \left(u\cdot \nabla \right)w_{k}=\nabla \cdot j_{k}+R_{k}$$ (4)

The electrostatic field is

$$\varepsilon_{0}\varepsilon_{r}\nabla \cdot E=\rho_{q}$$ (5)

The space charge density is

$$\rho_{q}=q\left(\sum_{k=1}^{N}Z_{k}n_{k}-n_{e}\right)$$ (6)

**Fig. 2.**
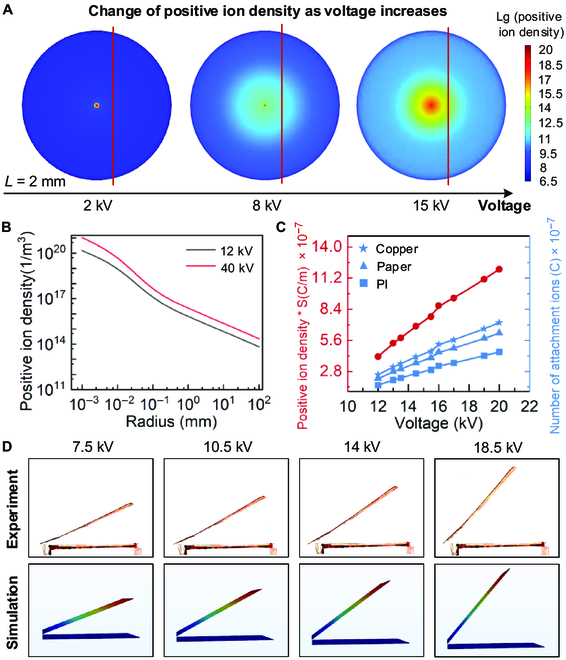
Theoretical analysis and simulation of single-electrode electrostatic repulsion phenomenon. (A) Simulation of single electrode subjected to different voltages. Lg, log_10_. (B) Positive ion density–radius curve of different voltages. (C) Attachment ion amount–voltage curve of different materials. (D) Experiments and simulations of deformation sheet.

By solving the above equation with COMSOL Multiphysics, we can obtain the distribution of positive ions in space for a specific voltage (see Fig. 2A and B). At lower voltages, corona zone is narrow, and there are hardly any free ions in the air; hence, electrostatic attraction is observed to predominate. As the voltage rises to between *U*_1_ and *U*_0_, the corona is gradually enhanced, and the object at a distance of 2 mm from the center gradually attaches some free ions at this time, which is the coexistence region. When the voltage rises above *U*_0_, a large number of free ions form around the electrode, at which point the corona dominates and the phenomenon takes on the characteristics of electrostatic repulsion.

Free positive ions will attach to the surface of the object in the ionized zone as the concentration of positive ions in the corona region increases throughout the ionization process. The property of the surface to which the object is attached has an impact on this attachment process. Since positive ion amount cannot be directly observed and measured, we combine experiments and simulations to calculate the positive ion amount attached to the deformation sheet to obtain the physical laws of the attachment process.

An object in an electric field is subjected to the phenomenon of electrostatic induction at the same time, and the electric field force on it is calculated by Coulomb’s law

$$F=\int E\left(q_{p}-q_{i}\right)$$ (7)

where *q_p_* is the charges of attached ion and *q_i_* is induced charges.

However, once the attachment process has progressed to a certain point, the electrostatic induction phenomenon becomes weak since there are far more positive ions attached than there are charges induced. As a result, the object in the electric field is primarily subject to the action of the Coulomb force produced by the attached ions in the electric field. By experimentally measuring the magnitude of the electric field force required for a specific deformation of the deformation sheet (more details can be found in the “Measurement of electric field force” section), the charges of attached ion amounts $q_{p}^{*}$ attached to the deformation sheet can be calculated as

$$q_{p}^{*}=\frac{{\mathrm{Fl}}_{1}\text{sin}\theta }{{\mathrm{El}}_{2}}$$ (8)

where *l*_1_ and *l*_2_ are the lengths of the force arms of the moments generated by the tension and electric field forces, respectively, and *θ* is the angle of deformation.

Since the attached ion amounts depend on the materials of the deformation sheet, we explored the attached ion amounts for some materials in Fig. S2. Figure 2C shows that as the voltage increases, the attached ion amounts on the deformation sheet gradually increase; the trend is the same for different materials, but there are variable numbers of attached ions. We define the attachment coefficient *k*, a quantity that depends on material and surface characteristics of the deformation sheet. Assuming that the deformation sheet has *N* surfaces composed of different materials, the electrostatic repulsion force generated by the single electrode can be described as

$$F=\sum_{j=1}^{N}k_{j}\rho_{q}S_{j}E$$ (9)

where *k_j_* and *S_j_* are the attachment coefficient and area of the *j*th surface, respectively.

We simulate the calculated attached ion amounts as a known quantity in COMSOL Multiphysics, and Fig. 2D shows that the simulation results agree well with the experiments, demonstrating that the theoretical model and calculated attached ion amounts are reliable and correct.

### Large deformation and fast actuation

A single-electrode tweezer is used for actuation, which requires only an electrode sheet and a deformation sheet, thereby eliminating the need for negative electrodes, avoiding dielectric breakdown and short circuit from the principle. In fact, dielectric breakdown and short circuit are the fatal defects of most existing electrostatic actuation methods [31–37], which greatly reduce the stability and reliability of the electrostatic actuators. We utilize a flapping wing (Fig. S3, Movie S4 and Materials and Methods-“Dielectric breakdown comparison of traditional actuation and electrostatic tweezer”) to visually show this feature. Wing driven by the 2-electrode electrostatic attraction actuation with positive and negative electrodes (traditional actuation) easily suffers dielectric breakdown, leading to failure. In contrast, electrostatic tweezer driven wing is capable of continuous operation without dielectric breakdown and maintains an excellent operating frequency. For the 2-electrode electrostatic attraction actuation, the deformation is small, while electrostatic tweezer can produce large deformation. This is because electrostatic attraction would fail at high voltage, while repulsion force would increase with voltage, resulting in a larger deformation. In addition, electrostatic tweezer has no special requirements for the materials of the driven objects. This means that when used to drive a machine, no need to arrange electrodes on the deformation sheet, thus reducing the constraint of the electrodes and wires to the motion and reducing the complexity of the structure designs.

**Fig. 3.**
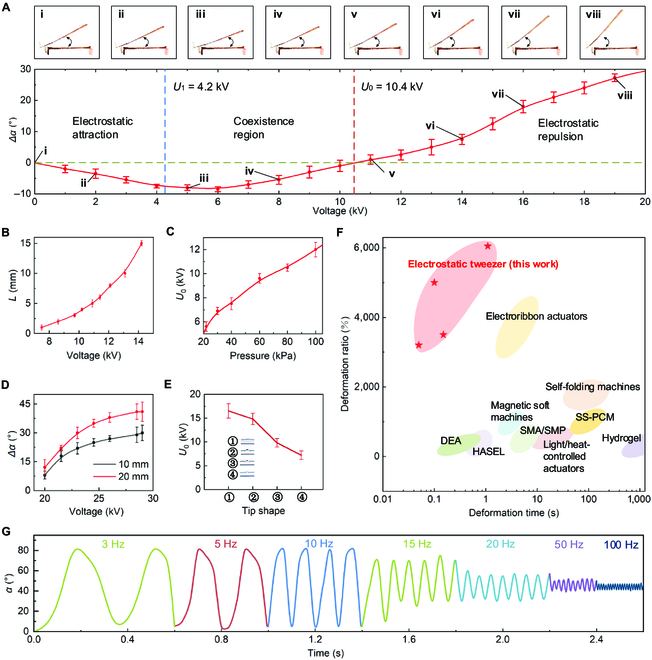
Characterization of electrostatic tweezer. (A) Bending angle versus voltage curve for electrostatic tweezer. *U*_1_ = 4.2 kV and *U*_0_ = 10.4 kV. (B) Influence of the distance between the electrode sheet and the deformation sheet on *U*_0_. (C) Influence of the air pressure on *U*_0_. (D) Influence of the relative area of the electrode sheet and the deformation sheet on deformation. (E) Influence of the shape of the electrode sheet on *U*_0_. (F) Comparison of deformation ratio and deformation time between electrostatic tweezer in this study and typical smart material actuation methods [S1–S14]. DEA, dielectric elastomer actuators; HASEL, hydraulically amplified self-healing electrostatic actuators; SMA/SMP, shape-memory alloy/shape-memory polymers; SS-PCM, shape-stabilized phase change material. (G) Deformation curves for electrostatic tweezer at applied voltages with an amplitude of 20 kV and frequencies ranging from 3 to 100 Hz.

To characterize the performance of electrostatic tweezer, we performed some quantitative tests. Fig. 3A depicts how the deformation sheet’s bending angle changes as the voltage gradually increases, where the inward bending angle is defined as the negative angle and the outward bending angle is defined as the positive angle. It can be seen that its *U*_1_ is ~ 4.2 kV and *U*_0_ is ~ 10.4 kV. For electrostatic tweezer, *U*_0_ is critical since the repulsion phenomenon only occurs when the voltage exceeds *U*_0_. The value of *U*_0_ influenced by the distance between the electrode sheet and the deformation sheet, the air pressure, the shape of the electrode sheet, etc. *U*_0_ increases with the distance (Fig. 3B and Fig. S4) which is due to the fact that the electric field weakens with distance, requiring higher voltages to produce a weak corona. *U*_0_ decreases as air pressure drops (Fig. 3C), which is related to the fact that air saturation is influenced by air pressure and that there is less air the easier it is to form the weak corona. The area of the electrode sheet has little effect on *U*_0_ (Fig. S5), but the larger the relative area of the electrode sheet and the deformation sheet after entering the repulsion region, the greater the repulsion force generated, which leads to larger deformation (Fig. 3D). The sharper the surface of the electrode sheet, the smaller the *U*_0_ (Fig. 3E), even down to 7 kV.

Electrostatic tweezer exhibits larger and faster actuations compared to typical smart materials actuation methods [S1–S14] (Fig. 3F and Table S1), enabling more than 60 times the deformation within 0.5 s. Electrostatic tweezer possesses high-speed actuation (Fig. 3G and Movie S5) and can still maintain a large deformation when the driving frequency reaches 20 Hz; its driving frequency can reach more than 100 Hz due to its excellent electrical response characteristics. Naturally, if the voltage increases further, the deformation angle will also increase. In addition, it is also possible to achieve larger deformation by improving the materials of the deformation sheet.

### Electrostatic tweezer for metamaterials and robots

**Fig. 4.**
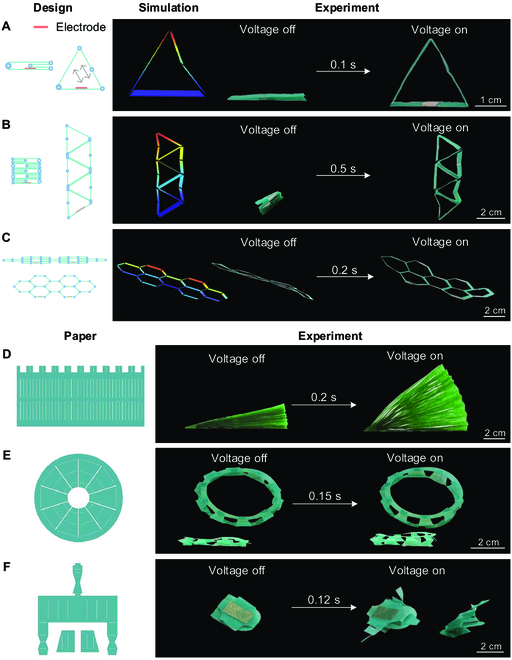
Electrostatic tweezer for metamaterials. (A) Triangular unit. (B) Triangular lattice. (C) Hexagonal lattice. (D) Fan shape. (E) Cylindrical shape. (F) Bird shape. The actuating electrodes are arranged at the bottom.

Electrostatic tweezer can produce repulsion force and achieve noncontact and remote actuation, with the advantages of large deformation, fast actuation, and simple structure, which is ideal for metamaterials and robots. We exhibited a series of metamaterials (Fig. 4, Fig. S6, and Movie S6) that were designed and fabricated using origami [41]. To guide the design process of metamaterials, we used the equivalent charges to analyze the deformation of the metamaterials through finite element simulation (Fig. S7). As shown in Fig. 4A to C, the finite element simulation predictions agree well with the experiment results, verifying the accuracy of the models. These metamaterials enable a 2-dimensional to 3-dimensional transformation. Because of the simplicity of the structures required for electrostatic tweezer, this 3-dimensional mode of metamaterials can be engineered into a variety of forms. When translating to 2-dimensional mode, the metamaterials can be compressed into thin, flat structures that occupy little space. The response time of deformation is related to the designed 3-dimensional structure. In our experiments, the response time of the structure with the highest deformation ratio takes less than 0.5 s, and the fastest reaches within 0.1 s (Fig. S8).

**Fig. 5.**
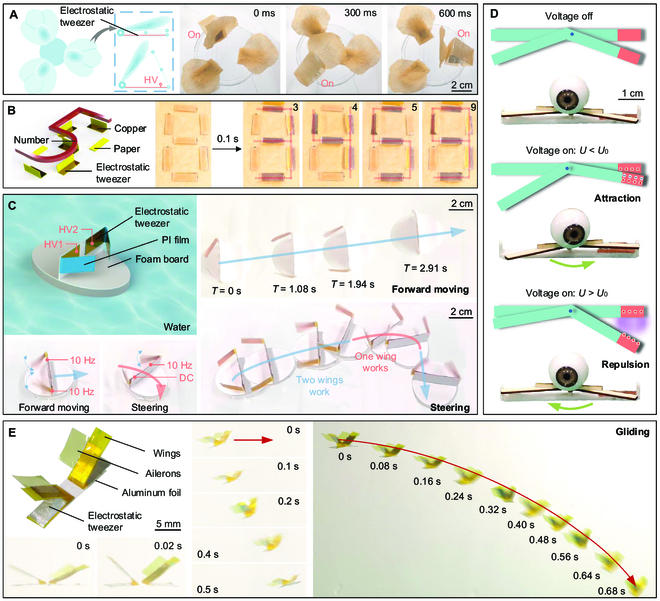
Electrostatic tweezer for robots. (A) Flower-shaped robot and its sequential actuation. (B) Digital display system. (C) Water surface robot and its forward moving and steering. (D) Robotic eyes. (E) Flapping-wing microscale aerial vehicle and its motions.

Electrostatic actuation is susceptible to interference from multiple electrode sheets. To demonstrate the anti-interference capability of electrostatic tweezer, we designed a flower-shaped robot using an electrostatic tweezer with 3 electrodes. The flower-shaped robot has 3 petals, and each petal is arranged with an actuation electrode sheet (Fig. 5A and the “Flower-shaped robot” section). Each petal can be rapidly actuated independently and sequentially controlled (Fig. 5A and Movie S7), demonstrating that electrostatic tweezer possesses certain anti-interference capability, excellent controllability, and independent control capability. In addition, on the basis of these features, we designed a digital display system using the electrostatic tweezer, which enables the display of numbers (Fig. 5B, Movie S8, and the “Digital display system” section). Moreover, we designed a water surface robot that is driven using an electrostatic tweezer with 2 electrodes (Fig. 5C and the “Water surface robot” section). This robot can achieve fast water glide speed (>1.6 body length/s) and controlled straight travel and turn (Fig. 5C and Movie S9), verifying that electrostatic tweezer has excellent actuation and control performance on mobile robots.

Electrostatic tweezer enables noncontact actuation and can switch between attraction and repulsion forces by adjusting the voltage. We applied electrostatic tweezer to the robotic eyes by applying different voltages to the base of the eyes to attract or repel the side of the eyes, allowing the robotic eyes to rotate counterclockwise or clockwise (Fig. 5D, Fig. S9, and Movie S10). Electrostatic-tweezer-driven robotic eyes achieve controlled bidirectional rotation with a simple structure by varying the voltages of a single-ended electrode. In addition, electrostatic tweezer can be used as a driving method for flapping-wing microscale aerial vehicles [42] due to its large deformation and fast actuation. As shown in Fig. 5E and Movie S11, electrostatic tweezer can generate sufficient flutter frequency and amplitude to drive the simple flapping-wing vehicle’s motion. The flapping-wing vehicle produces a horizontal motion when hung, as seen in Fig. 5E, illustrating the electrostatic tweezer’s capacity to drive in the horizontal direction. After cutting the hanging string, the flapping-wing vehicle can glide for some distance (Fig. 5E). Compared with previously reported metamaterials and robots [S2,S5,S15–S21] (Table S2), ours possess the advantages of simple structure, large deformation, fast deformation speed, lightweight, and easy arrangement of actuation, demonstrating electrostatic tweezer’s great application prospects in metamaterials and robots.

### Remote manipulation

**Fig. 6.**
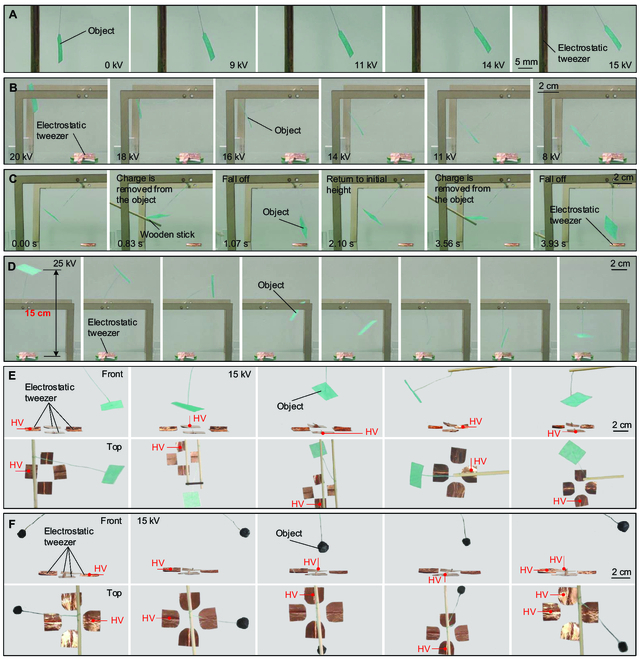
Remote manipulation. (A) A rod electrode as a simple electrostatic tweezer for manipulating sheet-like object. (B) A planar electrode as a simple electrostatic tweezer for manipulating sheet-like object. (C) Removal charge tests. (D) Manipulating distance of the electrostatic tweezer can reach up to 15 cm. (E) Electrostatic tweezer with 4 electrodes for manipulating sheet-like object. (F) Electrostatic tweezer with 4 electrodes for manipulating spherical object.

To exhibit the practical manipulation of electrostatic tweezers, we established an experimental setup for manipulating sheet-like and spherical objects that are frequently seen in scientific area. We used a rod electrode as a simple electrostatic tweezer for manipulating sheet-like objects. Electrostatic repulsion works when the voltage rises beyond the threshold voltage *U*_0_, allowing the sheet-like object to be manipulating to desired positions (Fig. 6A and Movie S12). To demonstrate the manipulation on a vertical plane, we use a planar electrode as a simple electrostatic tweezer. The manipulation procedure is shown in Fig. 6B and Movie S13, where it is evident that the sheet-like object can remain stable at a desired height. The sheet-like object will fall off when we use a wooden stick to remove the charge from it, but it will recharge in the electrostatic tweezers region and return to its initial height (Fig. 6C and Movie S14). This phenomenon is consistent with our explanation and theoretical model of single-electrode electrostatic repulsion. We further illustrate that the manipulating distance can reach up to 15 cm, and the object can be fixed steadily in this position (Fig. 6D and Movie S15).

To demonstrate multidimensional spatial manipulations, we constructed an electrostatic tweezer with 4 planar electrode plates. Through the manipulation of electrostatic tweezers, the sheet-like object can remain in a fixed position in the air (Fig. 6E and Movie S16). In addition to the sheet-like object, we also used electrostatic tweezers to manipulate the spherical object (Fig. 6F and Movie S17). As soon as the voltage is applied, the spherical object will be quickly pushed away and then stabilized. When the density of the electrode arrangement in the electrostatic tweezers is higher and the space is enlarged, the spatial position of the manipulated object will be greater. Compared to optical tweezers [13,18,19], electrostatic tweezers can be used to manipulate macroscopic objects. Compared to acoustic tweezers [14,20,21], electrostatic tweezers do not require closed spaces and are simple to install. Compared to magnetic actuation [15–17,22,23], electrostatic tweezers have a larger range of manipulation objects, which can be either conductive or nonconductive. Compared to existing electrostatic tweezers (Table S3) [30,43], our electrostatic tweezers have the advantage of long manipulation distance. Therefore, electrostatic tweezers can be used as a simple device for remote manipulation of various macroscopic objects in real-world circumstances.

## Discussion

We have reported a new phenomenon in electrostatics—single-electrode electrostatic repulsion phenomenon—and demonstrated the existence of 3 regions including electrostatic attraction region, coexistence region, and electrostatic repulsion region. Through theoretical analysis, simulation, and experiment methods, the phenomenon of single-electrode electrostatic repulsion is explained. On the basis of this finding, we have proposed the concept of electrostatic tweezers, which can achieve noncontact and remote actuation and manipulation. In addition, we have demonstrated that electrostatic tweezers can produce large deformation rates (>6,000%), fast actuation (>100 Hz), and remote manipulation distance (~15 cm) and have the advantages of simple device structure, easy control, lightweight, no dielectric breakdown, and low cost. Briefly, the single-electrode electrostatic repulsion phenomenon and electrostatic tweezers here offer a new platform for exploring and applying electrostatics and open previously unexplored opportunities for remote actuation and manipulation various macroscopic objects in real-world circumstances using electrostatics.

## Materials and Methods

### Basic test setup

The basic test setup (Fig. S1) consisted of a single-electrode sheet (electrostatic tweezer), a deformation sheet (object), and an acrylic base. The acrylic plate base was created by laser cutting a 3-mm-thick acrylic plate into the desired shape. A 50-μm copper foil tape was placed onto an acrylic board to create the single-electrode sheet. The deformation sheet was made by adhering a copper foil tape with a thickness of 50 μm to a polyimide (PI) film with a thickness of 50 μm. To avoid the direct contact of the single-electrode sheet and the deformation sheet during the electrostatic attraction stage, thereby causing charge transfer, the single-electrode sheet and the deformation sheet were not parallel but at a certain angle in the initial state. The basic test setup is placed on a 30-cm-high acrylic stand. The basic test setup is electrically connected through a single wire and otherwise does not come into contact with anything.

### Vacuum test

The phenomenon of corona is quite obvious in an environment with thin air. The electrostatic test setup was placed in a sealed transparent box. First, the electrostatic test setup was energized to generate electrostatic exclusion, and then the box was gradually vacuumed. In this process, an obvious glow phenomenon can be observed on the electrostatic test setup (Movie S18). This proves that the weak corona is the cause of the single-electrostatic repulsion phenomenon and leads to the charge on the deformation sheet.

### Measurement of electric field force

We use an indirect measurement method to measure the electric field force. Once the deformation sheet has been observed to have deformed, we pull it at its center of mass location using a force gauge. We then record the force needed for the deformation sheet to deform to the same angle, which is thought to be the force produced by the electric field.

### Dielectric breakdown comparison of traditional actuation and electrostatic tweezer

The device used in the comparison experiment for dielectric breakdown was a flapping wing structure (Fig. S3). The flapping wing structure consisted of 2 arc-shaped bases and a deformation sheet. Two arc-shaped bases were created from 2 pieces of acrylic board using laser cutting. Copper foil was pasted on the inner side of the arc-shaped base to form the single-electrode sheet. The deformation sheet was formed by pasting the cut copper foil onto the PI film.

In electrostatic tweezer control, a high voltage of a certain frequency was given to the single-electrode sheet, which created repulsion force to push the deformation sheet to move, producing flapping resembling a dragonfly’s wings. When electrostatic attraction was used to control the structure, the deformation sheet was grounded, and a high voltage at a specific frequency was applied to the single-electrode sheet to generate attraction.

### Switching voltage threshold *U*_0_

To determine the switching voltage threshold *U*_0_, we gradually increased the voltage and observe the movement of the deformation sheet. As the voltage increased, the deformation sheet was first adsorbed and then repelled. Record the voltage value of the deformation sheet back to its original position. Then, we directly applied the recording voltage value to the electrostatic tweezer to observe the behavior of the deformation sheet. This voltage value would be regarded as the switching voltage threshold if the deformation sheet is not immediately adsorbed or repelled.

### Experimental design of the influence of the distance on *U*_0_

The structure of the experimental setup adopted the design of the basic test setup. The electrostatic tweezer was arranged parallel to the deformation sheet. It is possible to determine whether the force on the deformation sheet was attraction or repulsion by looking at the deformation sheet’s initial movement direction. The voltage was gradually changed, while the distance between the electrostatic tweezer and the deformation sheet was fixed. The voltage at which the repulsion force first appeared was referred to as the switching voltage threshold for the distance. In this way, we could determine the switching voltage threshold at several specific distances to illustrate the relationship between distance and the switching voltage threshold.

### Experimental design of the relationship between *U*_0_ and air pressure

The air pressure has an impact on the switching voltage threshold since it affects how the corona develops. The basic setup was positioned in a bigger vacuum box for the duration of the testing. We measured the switching voltage threshold as the device stabilized at a particular vacuum level. To reduce mistakes, the vacuum box equipment was brought back to the state of atmospheric pressure after every measurement.

### Electrostatic tweezer drives various materials

The experiment setup driving with various materials consisted of a base and various material samples, where material samples were mounted on the base through bearings (Fig. S2). Material samples included wood chip samples (balsa wood with a thickness of 1.5 mm), metal samples (one side of a strip of copper foil was pasted on the wood chip base, and the other side acts as the driven side), PI samples, and paper samples (these 2 are similar to metal samples, and only the material was replaced). The base was 3-dimensionally printed by fused deposition modeling, and the printed material was polylactic acid. A 50-μm-thick copper foil tape was pasted on the plane of the base, and a copper tip was set apart to boost the ionization ability to improve the driving ability.

### Fabrication and simulation of metamaterials

The production process of metamaterials (Fig. 4 and Fig. S6) was as follows: First, determine the final 3-dimensional structure, disassemble it into 3-dimensional modules, and then reverse-design the required 2-dimensional structure. The soft material was cut into the required 2-dimensional structure by laser cutting, and a 50-μm aluminum foil tape was pasted on the place where it needs to be driven. The required 3-dimensional structure was formed by origami and pasted with glue. Paper with a thickness of 50 μm was used in the experiment as the soft material.

To guide the design process of the metamaterials, we used the equivalent charges to analyze the deformation of the metamaterials through finite element simulation (Fig. S7). As shown in Fig. 4 and Fig. S7, the finite element simulation predictions agree well with the experiment results, verifying the accuracy of the models.

### Flower-shaped robot

A flower-shaped robot (Fig. 5A) consisted of an acrylic base and a number of petal modules. A single petal module assembled from a drive structure and a paper petal that had been adhered to the drive structure. The driving structures were made in a way similarly to the deploying structures. Paper with a thickness of 50 μm was used to press the petals.

### Digital display system

The digital display system (Fig. 5B) consisted of a wooden base and folded paper sheets arranged in an array. The folded paper was pasted at a specific position on the base, and the wires were connected through the through holes arranged on the base. By applying high voltage to different wires, the control of specific folded papers was realized.

### Water surface robot

The structure of a water surface robot (Fig. 5C) consisted of a base, a flapping wing support, and 2 flapping wings. The material of the base was a foam, which was laser-cut into an oval shape. The material of the flapping wings’ support was a cardboard, which was formed by laser cutting and then shaped by folding. The PI film was sliced into strips, and copper foil was then adhered to both sides of each strip. One side was set to be the electrostatic tweezer, and the other side was the deformation sheet. The strips were folded from the middle of the strips. Because of the plastic deformation, the PI strips would maintain a bending angle after folding, leaving enough space between 2 pieces of copper foil so that they do not touch after plastic deformation.

### Robotic eye

The robotic eye (Fig. 5D) consisted of a base and an eyeball structure (Fig. S9). The base was formed by fused deposition modeling 3-dimensional printing, and the material was polylactic acid. The main body of the eyeball was made of acrylonitrile butadiene styrene plastic. The paulownia board was laser-cut and glued to the back of the eyeball for force transmission. The base and the eyeball structure were connected through bearings. The copper foil was pasted on the same side of the base and the paulownia board; the copper foil on the base was used as the fixed end, and the copper foil on the paulownia board was used as the floating end.

### Flapping-wing microscale aerial vehicle

A flapping-wing microscale aerial vehicle (Fig. 5E) consisted of a paper base, 2 wings made of 50-μm PI film, and 2 ailerons made of 6-μm PI film. The aluminum films were pasted on the inside of the wings, and the ailerons were pasted on the wings.

## Data Availability

The data that support the findings of this study are available in the main text and the Supplementary Materials. The data are also available from the corresponding authors upon request.

## Supplementary Materials

Supplementary 1 Figs. S1 to S9 Tables S1 to S3 Movies S1 to S18 Supplementary References
