# Supplementary material for: Single-Electrode Electrostatic Repulsion Phenomenon for Remote Actuation and Manipulation
Source: Research (Wash D C). 2024 May 29;7:0393. doi: 10.34133/research.0393 (PMC11134173; doi:10.34133/research.0393)
Supplement: Supplementary 1 — Figs. S1 to S9 Tables S1 to S3 Movies S1 to S18 Supplementary References [file research.0393.f1.zip › Supplementary Materials.pdf]

Supplementary Materials

Single-electrode electrostatic repulsion phenomenon for remote actuation and manipulation

Wei Tang et al.

Correspondence to: junzou@zju.edu.cn

This PDF file includes:

Fig. S1. Basic test setup
Fig. S2. Electrostatic tweezer is used to drive various materials
Fig. S3. A Two-electrode electrostatic attraction actuation (traditional actuation) and electrostatic tweezer for driving flapping wing systems
Fig. S4. Set up for the influence of the distance on the switching voltage threshold
Fig. S5. The influence of the relative area of the electrode sheet surface on U_0
Fig. S6. Papers for metamaterials
Fig. S7. Finite element analysis simulation of metamaterials
Fig. S8. Response time of electrostatic tweezer under deformation of a triangular metamaterial
Fig. S9. The structure of the robotic eye
Table S1. Comparison of deformation ratio and deformation time between electrostatic tweezer and typical smart materials actuation methods
Table S2. Comparison of various metamaterials and robots
Table S3. Comparison of different electrostatic tweezers
References (S1–S23)
Captions for movies S1 to 18

Other Supplementary Materials for this manuscript include the following:

Movie S1. Single-electrode electrostatic repulsion compared with electrostatic induction
Movie S2. The phenomena of single-electrode electrostatic repulsion and electrostatic induction occur continuously
Movie S3. Electrostatic tweezer is used to drive various materials
Movie S4. Comparison of dielectric breakdown characteristics between traditional actuation and electrostatic tweezer
Movie S5. Frequency tests of electrostatic tweezer
Movie S6. Electrostatic tweezer for metamaterials
Movie S7. Electrostatic tweezer for a flower-shaped robot

Movie S8. Electrostatic tweezer for a digital display system
Movie S9. Electrostatic tweezer for a water surface robot
Movie S10. Control the robotic eye via attraction and repulsion forces
Movie S11. Electrostatic tweezer for a flapping-wing microscale aerial vehicle
Movie S12. A rod electrode as a simple electrostatic tweezer for manipulating sheet-like object
Movie S13. A planar electrode as a simple electrostatic tweezer for manipulating sheet-like object
Movie S14. Removal charge tests
Movie S15. Manipulating distance of the electrostatic tweezer can reach up to 15 cm
Movie S16. Electrostatic tweezer with four electrodes for manipulating sheet-like object
Movie S17. Electrostatic tweezer with four electrodes for manipulating spherical object
Movie S18. Vacuum test

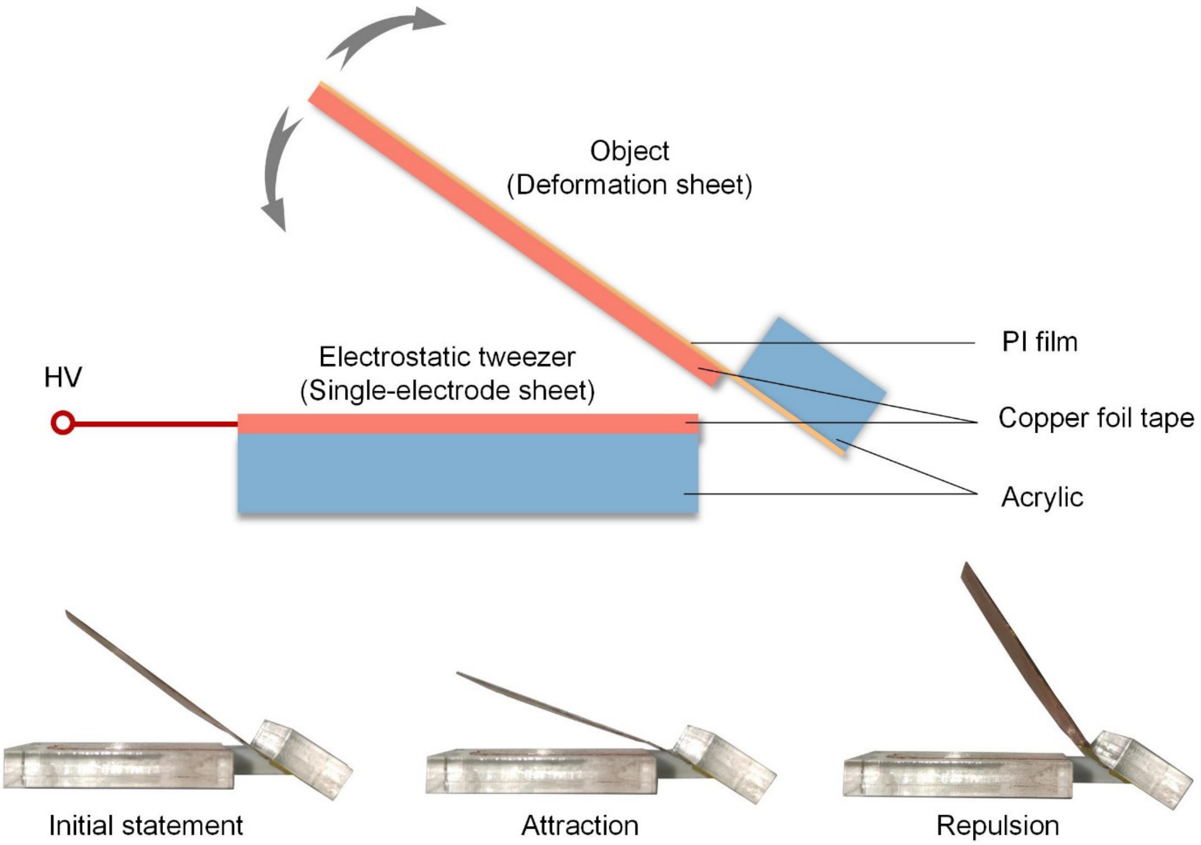

Fig. S1. Basic test setup. A single-electrode sheet (electrostatic tweezer) and a deformation sheet (object), which are each mounted to the base of the acrylic board, are the major components of the basic test setup. The deformation sheet is made by pasting copper foil tape on the PI film. The copper foil tape directly applied to the acrylic board base is used as the single-electrode sheet. Apply high voltage to the single-electrode sheet during the test to observe if the floating end's angle changes. The initial angle is defined as 0, the inward bending angle is defined as the negative angle, and the outward bending angle is defined as the positive angle.

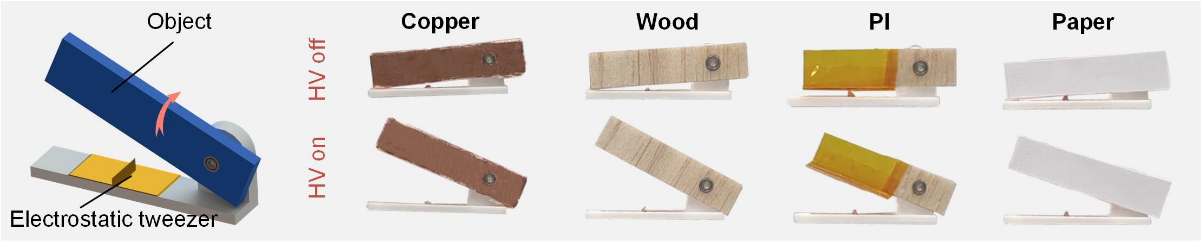

Fig. S2. Electrostatic tweezer is used to drive various materials. Copper, wood, PI (Polyimide), and paper.

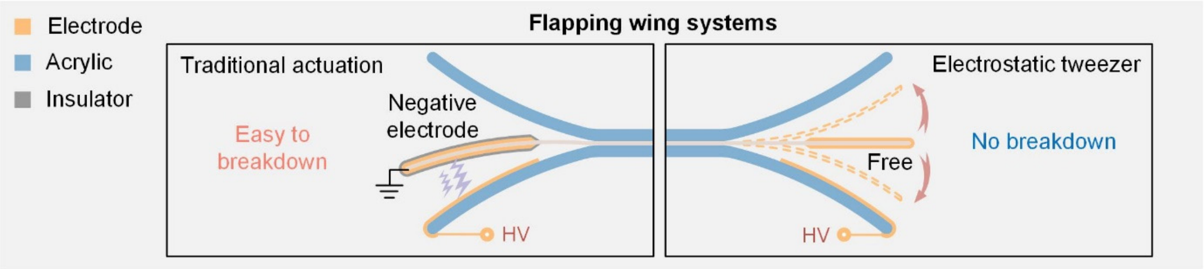

Fig. S3. A Two-electrode electrostatic attraction actuation (traditional actuation) and electrostatic tweezer for driving flapping wing systems.

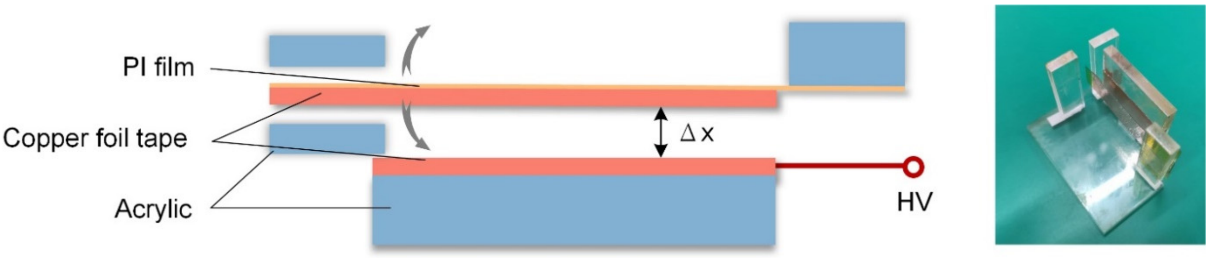

Fig. S4. Set up for the influence of the distance on the switching voltage threshold. It uses the same components and fabrication ways as the basic test setup. The distance between a single-electrode sheet (electrostatic tweezer) and a deformation sheet is set to the value to be measured. The deformation sheet has two acrylic plate at both side of its end to make it easier to determine the force's direction.

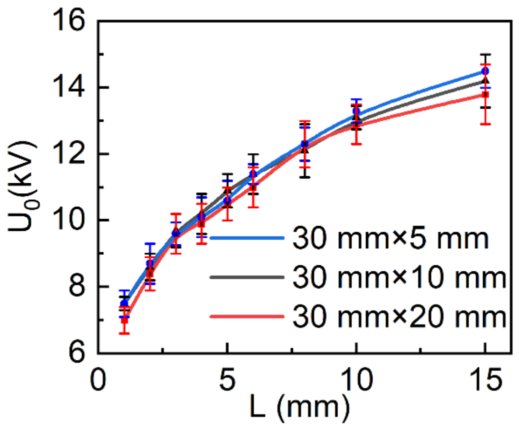

Fig. S5. The influence of the relative area of the electrode sheet surface on U_0. A single-electrode sheet and a deformation sheet have a parallel relative maximum area. The U_0 of electrodes with different relative areas at various distances is determined in this experiment. Three groups of electrode sheets, each 30 mm by 5 mm, 30 mm by 10 mm, and 30 mm by 20 mm, were utilized in the experiment. The results of the experiment show the relative area of the electrode sheet surface has little effect on U_0.

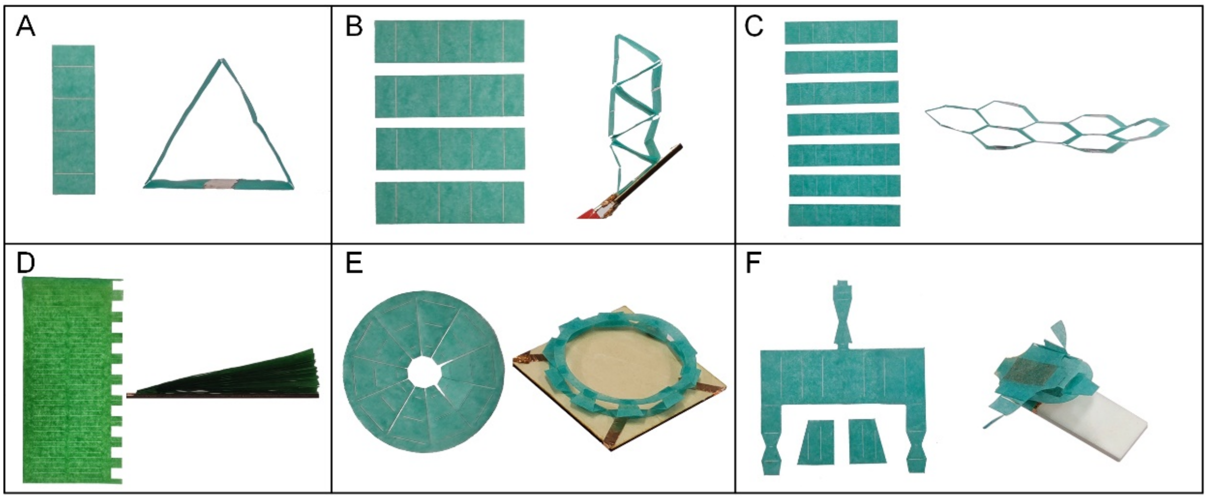

Fig. S6. Papers for metamaterials. Through the art of origami, metamaterials can be created from two-dimensional materials. The image displays the two-dimensional, initial form of the metamaterials described in the article. These structures are folded to create various metamaterials.

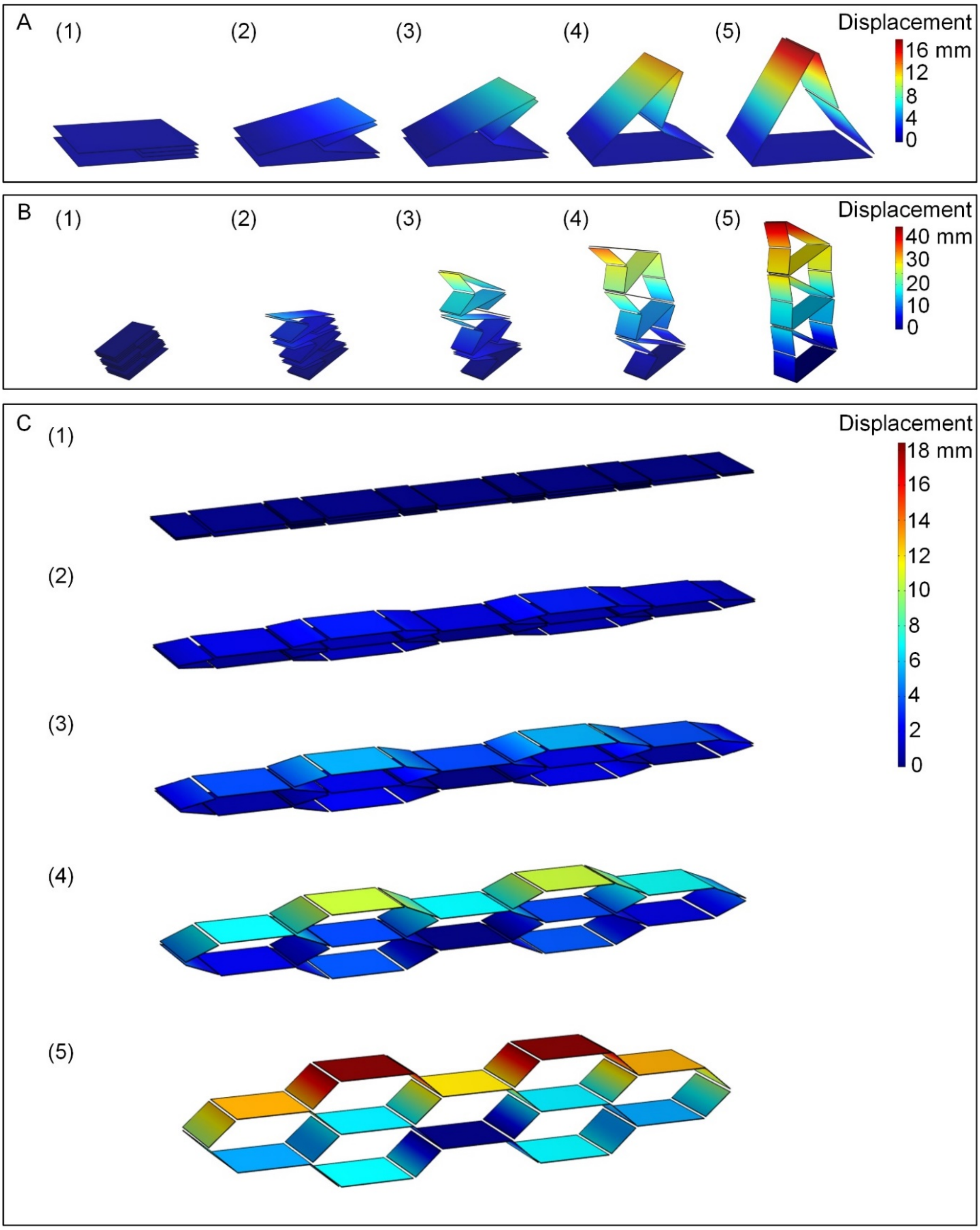

Fig. S7. Finite element analysis simulation of metamaterials. Comsol Multiphysics is used for the simulation process. The equivalent charges are used to analyze the deformation of the metamaterials through finite element simulation. The electrostatic field and multi-body kinematics are coupled, and the flexible hinge of the physical object is roughly represented as a hinge constraint in the simulation. The simulation results agree with the experiments.

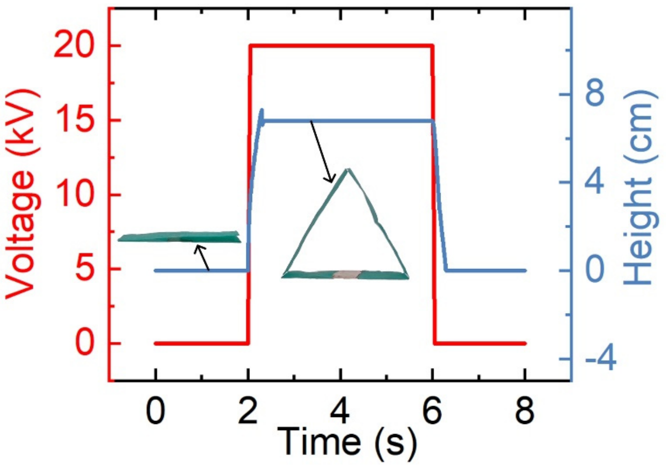

Fig. S8. Response time of electrostatic tweezer under deformation of a triangular metamaterial. As a driving method, Electrostatic tweezer has different response curves for different structures but has similar properties. Here, the deformation of the triangular metamaterial is taken as an example to show the response of electrostatic tweezer under the step signal.

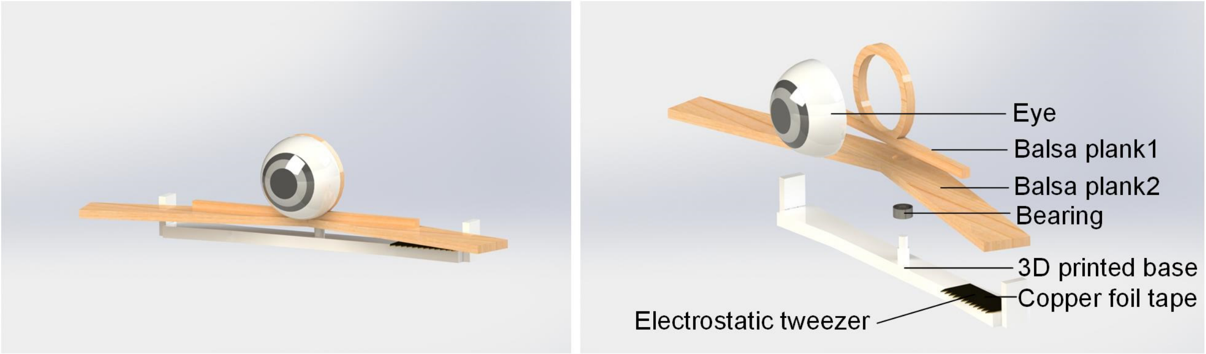

Fig. S9. The structure of the robotic eye. The prosthetic eye is glued to balsa plank 1, and then the entire thing is glued to the specified position of balsa plank 2. Through bearings, the entire paste is attached to the 3D-printed base. Copper foil tape should be applied to one side of the 3D-printed base. The robotic eyes are driven to swing by applying different voltages to the electrostatic tweezer to generate repulsive and attraction forces, respectively.

Table S1. Comparison of deformation ratio and deformation time between electrostatic tweezer and typical smart materials actuation methods

	Deformation ratio	Deformation time
Hydraulically amplified self-healing electrostatic actuators (HASEL) [S1]	~ 130–224%	~ 0.5–1.2 s
Electro-ribbon actuators [S2]	~ 600–3500%	~ 2–6 s
Magnetic soft machines [S3,S4]	~ 500–1200%	~ 3–5 s
Self-folding machines [S5]	~ 1000–2100%	~ 50–80 s
Light/heat-controlled actuators [S6,S7]	~ 150–520%	~ 10–180 s
Hydrogel [S8,S9]	~ 120–140%	~ 300–500 s
Dielectric elastomer actuator (DEA) [S10,S11]	~ 110–320%	~ 0.1–1 s
Shape-memory alloy/ Shape-memory polymers (SMA/SMP) [S12]	~ 300–1100%	~ 1–5 s
Shape-stabilized phase change material (SS-PCM) [S13,S14]	~ 290–980%	~ 60–100 s
**Electrostatic tweezer (this study)**	**~ 3000–6000%**	**~ 0.1–0.6 s**

Note: The values listed in the table are estimated values which are measured from the pictures or videos given in the references. The deformation ratio refers to the ratio of the maximum length in a single direction to the initial length during the deformation process of the structure.

Supplementary Table 2. Comparison of various metamaterials and robots

	Weight	Deformation ratio	Deformation time	Fabrication	Recover	Design
Self-folding machines [S5]	Heavy	~ 1000–2000%	> 60 s	Complex	No	Diverse
Electro-origami robots [S2]	Light-weight	~ 600–3500%	~ 2–6 s	Easy	Yes	Limited
Robotic metamorphosis by origami exoskeletons [S15]	Light-weight	~ 10–30%	> 60 s	Easy	No	Diverse
Soft robotic origami crawler [S16]	Light-weight	~ 40–90%	~ 0.2–1 s	Easy	Yes	Limited
Ultrathin thermoresponsive self-folding 3D graphene [S17]	Light-weight	~ 500–1000%	~ 2–4 s	Complex	Yes	Diverse
An End-to-End Approach to Self-Folding Origami Structures [S18]	Heavy	~ 800–1000%	> 60 s	Complex	No	Diverse
Origami-Based Reconfigurable Metamaterials [S19]	Light-weight	~ 20–40%	\	Complex	Yes	Limited
Self-Folding Origami by Pre-Biasing Vertex Buckling Direction [S20]	Light-weight	~ 300–600%	> 60 s	Complex	No	Diverse
Programmable Paper-Elastomer Composites [S21]	Heavy	~ 50–300%	~ 3–20 s	Complex	Yes	Diverse
**Electrostatic tweezer for metamaterials and robots (this study)**	**Light-weight**	**~ 3000–6000%**	**~ 0.1–0.6 s**	**Easy**	**Yes**	**Diverse**

Note: The values listed in the table are estimated values which are measured from the pictures or videos given in the references. The deformation ratio refers to the ratio of the maximum length in a single direction to the initial length during the deformation process of the structure.

Table S3. Comparison of different electrostatic tweezers

Electrostatic tweezer	Working principle	Manipulation distance
Triboelectric wetting for continuous droplet transport [S22]	Triboelectric wetting effect	~ 2 mm
Electrostatic tweezer for droplet manipulation [S23]	Electrostatic induction	~ 4 mm
**This study**	**Single-electrode electrostatic repulsion**	**~ 15 cm**

Supplementary Movie

Movie S1. Single-electrode electrostatic repulsion compared with electrostatic induction

Movie S2. The phenomena of single-electrode electrostatic repulsion and electrostatic induction occur continuously

Movie S3. Electrostatic tweezer is used to drive various materials

Movie S4. Comparison of dielectric breakdown characteristics between traditional actuation and electrostatic tweezer

Movie S5. Frequency tests of electrostatic tweezer

Movie S6. Electrostatic tweezer for metamaterials

Movie S7. Electrostatic tweezer for a flower-shaped robot

Movie S8. Electrostatic tweezer for a digital display system

Movie S9. Electrostatic tweezer for a water surface robot

Movie S10. Control the robotic eye via attraction and repulsion forces

Movie S11. Electrostatic tweezer for a flapping-wing microscale aerial vehicle

Movie S12. A rod electrode as a simple electrostatic tweezer for manipulating sheet-like object

Movie S13. A planar electrode as a simple electrostatic tweezer for manipulating sheet-like object

Movie S14. Removal charge tests

Movie S15. Manipulating distance of the electrostatic tweezer can reach up to 15 cm

Movie S16. Electrostatic tweezer with four electrodes for manipulating sheet-like object

Movie S17. Electrostatic tweezer with four electrodes for manipulating spherical object

Movie S18. Vacuum test

Supplementary References

S1. Acome, E. et al. Hydraulically amplified self-healing electrostatic actuators with muscle-like performance. *Science* **359**, 61-65 (2018).

S2. Taghavi, M., Helps, T. & Rossiter, J. Electro-ribbon actuators and electro-origami robots. *Sci. Robot.* **3**, eaau9795 (2018).

S3. Alapan, Y., Karacakol, A. C., Guzelhan, S. N., Isik, I. & Sitti, M. Reprogrammable shape morphing of magnetic soft machines. *Sci. Adv.* **6**, eabc6414 (2020).

S4. Song, H. et al. Reprogrammable Ferromagnetic Domains for Reconfigurable Soft Magnetic Actuators. *Nano Lett.* **20**, 5185-5192 (2020).

S5. Felton, S., Tolley, M., Demaine, E., Rus, D. & Wood, R. A method for building self-folding machines. *Science* **345**, 644-646(2014).

S6. Hou, K. et al. Programmable light-driven swimming actuators via wavelength signal switching. *Sci. Adv.* **7**, eabh3051 (2021).

S7. Yang, S. Y. et al. A jumping shape memory alloy under heat. *Sci. Rep.* **6**, 21754 (2016).

S8. Hu, L. X. et al. Hydrogel-Based Flexible Electronics. *Adv. Mater.* 2205326 (2022).

S9. Ko, J. K. et al. High-performance electrified hydrogel actuators based on wrinkled nanomembrane electrodes for untethered insect-scale soft aquabots. *Sci. Robot.* **7**, eabo6463 (2022).

S10. Gu, G. Y., Zou, J., Zhao, R. K., Zhao, X. H. & Zhu, X. Y. Soft wall-climbing robots. *Sci. Robot.* **3**, eaat2874 (2018).

S11. Li, G. R. et al. Self-powered soft robot in the Mariana Trench. *Nature* **591**, 66 (2021).

S12. Xu, L. et al. Locomotion of an untethered, worm-inspired soft robot driven by a shape-memory alloy skeleton. *Sci. Rep.* **12**, 12392 (2022).

S13. Boyvat, M., Vogt, D. M. & Wood, R. J. Ultrastrong and High-Stroke Wireless Soft Actuators through Liquid-Gas Phase Change. *Adv. Mater. Tech.* **4**, 1800381 (2019).

S14. Zhong, Y. D. et al. Programmable thermochromic soft actuators with "two dimensional" bilayer architectures for soft robotics. *Nano Energy* **102**, 107741 (2022).

S15. Miyashita, S., Guitron, S., Li, S. G. & Rus, D. Robotic metamorphosis by origami exoskeletons. *Sci. Robot.* **2**, eaao4369 (2017).

S16. Ze, Q. J. et al. Soft robotic origami crawler. *Sci. Adv.* **8**, eabm7834 (2022).

S17. Xu, W. A. et al. Ultrathin thermoresponsive self-folding 3D graphene. *Sci. Adv.* **3**, e1701084 (2017).

S18. An, B. et al. An End-to-End Approach to Self-Folding Origami Structures. *IEEE Trans. Robot.* **34**, 1409-1424 (2018).

S19. Wang, Z. J. et al. Origami-Based Reconfigurable Metamaterials for Tunable Chirality. *Adv. Mater.* **29**, 1700412 (2017).

S20. Kang, J. H., Kim, H., Santangelo, C. D. & Hayward, R. C. Enabling Robust Self-Folding Origami by Pre-Biasing Vertex Buckling Direction. *Adv. Mater.* **31**, 1903006 (2019).

S21. Martinez, R. V., Fish, C. R., Chen, X. & Whitesides, G. M. Elastomeric Origami: Programmable Paper-Elastomer Composites as Pneumatic Actuators. *Adv. Funct. Mater.* **22**, 1376-1384(2012).

S22. Xu. W., et al. Triboelectricwettingfor continuousdroplet transport. *Sci. Adv.* **8**, eade2085 (2022).

S23. Jin Y., et al. Electrostatic tweezer for droplet manipulation. *Proc. Natl. Acad. Sci. U.S.A.* **119**, e2105459119 (2022).
